# A simple setup for *in situ* alkali metal electronic spin polarimetry

**DOI:** 10.1063/5.0101537

**Published:** 2022-09-12

**Authors:** M. Kelley, R. T. Branca

**Affiliations:** Department of Physics and Astronomy, University of North Carolina at Chapel Hill, Chapel Hill, North Carolina 27514, USA

## Abstract

Faraday rotation is considered a gold standard measurement of the electronic spin polarization of an alkali metal vapor produced under optical pumping. However, during the production of large volumes of hyperpolarized xenon gas, transmission monitoring measurements, otherwise known as field cycling measurements, are generally employed to measure the spin polarization of alkali metal atoms *in situ* as this method is easier to implement than Faraday rotation on standard polarizer setups. Here, we present a simple, low-cost experimental setup to perform Faraday rotation measurements of the electronic spin polarization of alkali metal atoms that can be easily implemented on standard polarizer setups. We then compare Rb polarization measurements obtained with the Faraday rotation method to those obtained with the transmission monitoring method. To our knowledge, a direct comparison of these methods has never been made. Overall, we found good agreement between the two methods, but at low Rb density and high laser power, we found evidence of nonlinear magneto-optical effects that may prevent Faraday rotation from being used under these conditions.

## I. INTRODUCTION

The nuclear spin polarization of noble gases can be increased with respect to thermal polarization by several orders of magnitude using a process called spin-exchange optical pumping (SEOP). The first step of SEOP is optical pumping, where circularly polarized light produced by a high power laser is used to polarize the electronic spin of an alkali metal vapor. Then under spin-exchanging interactions, the electronic spin polarization of the alkali metal is transferred to the nuclear spins of noble gas atoms. As the final noble gas nuclear polarization depends on the density of the alkali metal vapor and on its electronic spin polarization, optimization of these parameters is generally needed to optimize the final noble gas polarization. However, the alkali metal polarization, specifically that of Rb, is too often assumed to be very close to unity, which contributed to a long standing discrepancy between the theoretically predicted and experimentally obtained nuclear polarization of ^129^Xe.^1,2^ The optimization of Rb polarization is even more important when using tunable, spectrally narrowed lasers capable of producing hundreds of watts as the amount of unused light dumped into the system can easily destabilize it and lead to Rb runaway.^3^ In addition, as there is conflicting evidence that off-resonance optical pumping can serve to increase Rb polarization^4^ and evidence that off-resonance pumping is limited by reduced circular dichroism,^5,6^ it is important to be able to monitor Rb polarization to optimize the pump wavelength experimentally.

Here, we report two methods of measuring the alkali metal polarization *in situ*: Faraday rotation, generally recognized as the gold standard for measuring Rb polarization, and transmission monitoring, which poses as a less robust but more adaptable method during the production of large volumes of hyperpolarized noble gases. To our knowledge, these methods have never been directly compared before. We describe a simple experimental setup for these two measurements and examine their feasibility and limitations for determining the alkali spin polarization during SEOP. A detailed description of the setup along with the bill of material is provided as Supplementary Material.

### A. Faraday rotation

The classical Faraday effect is a magneto-optical phenomenon in which a medium develops circular birefringence (i.e., the medium responds differently to left and right circularly polarized light) when a magnetic field is applied along the direction of light propagation. This is manifested in a different index of refraction for left and right circularly polarized light. When linearly polarized light propagates through the medium, the difference in the refractive index experienced by its left and right circularly polarized components causes a phase difference between the two components, which results in an apparent rotation of the light polarization known as the Faraday rotation angle, *θ*_*B*_. The Faraday rotation angle depends on the density of the medium, [*Rb*], the magnetic field strength, *B*, the path length of the probe light, *l*, and the detuning of the probe light from resonance, Δ,

$$\theta_{B}=\left[Rb\right]\frac{le^{2}\mu_{B}B}{18mhc}\left(\frac{4}{\Delta_{1/2}^{2}}+\frac{7}{\Delta_{3/2}^{2}}-\frac{2}{\Delta_{1/2}\Delta_{3/2}}\right),$$ (1)

where *e* is the electronic charge, *μ*_*B*_ is the Bohr magneton, m is the electron mass, *h* is the Planck constant, and *c* is the speed of light.^7^ To measure the density of a medium similar to that of an alkali vapor, the Faraday rotation method generally requires the use of large magnetic fields on the order of thousands of Vliegen.^8^

In the case of an optically pumped alkali vapor, the dominant Faraday rotation is no longer magnetic field dependent but depends on the electronic spin polarization, *P*_*Rb*_. This is typically referred to as the paramagnetic Faraday rotation,^7,9^ where

$$\theta_{P}=\left[Rb\right]\frac{le^{2}}{6mc}\left(\frac{\Delta_{3/2}}{{\left(\Delta_{3/2}-\delta_{3/2}\right)}^{2}}-\frac{\Delta_{1/2}}{{\left(\Delta_{1/2}-\delta_{1/2}\right)}^{2}}\right)P_{Rb}.$$ (2)

Here, we have included the dependence on the resonance linewidths, *δ*, which results in a small correction when the probe beam is adequately detuned from resonance. Note that Eqs. (1) and (2) are in Gaussian units. A nice derivation of these expressions can be found in the work by Kadlecek *et al*.^10^

It is common practice to use field dependent Faraday rotation, *θ*_*B*_, to measure the alkali density and, under optical pumping, to use paramagnetic Faraday rotation, *θ*_*P*_, to measure the electronic spin polarization. However, the experimental conditions used during SEOP are not ideal for measuring the alkali density using the field dependent Faraday rotation. Indeed, the magnetic fields used during SEOP are typically produced using electromagnetic coils, which can generate fields only on the order of tens of Gauss. In addition, the buffer gas pressures used during SEOP require substantial detuning from resonance to avoid probe absorption, again lowering the maximum Faraday rotation angle that can be generated. These issues make it untenable to measure the alkali densities typically obtained in optical cells during SEOP, which range from 10^17^–10^20^ m^−3^. For example, Vliegen *et al.*^8^ obtained successful measurement of an alkali atom density of 10^22^ m^−3^ in a 2 cm long optical cell only by using a magnetic field of 12 000 G. In the study by Mori *et al.*,^11^ it was noted that even at 3500 G, density measurements were only feasible when [*Rb*] × *l* was over 10^13^ atoms/cm^2^. Lock-in detection methods can be used to improve the measurement signal to noise ratio (SNR) but not to a degree that reduces the error enough for a reasonable measurement, especially for current SEOP setups. We even found that the changing capacitance of our photodiodes with incident light coupled with a pre-amplifier was enough to distort the output of our lock-in amplifier. Please see the supplementary material for more details. For these reasons, in what follows, we will only be using paramagnetic Faraday rotation to measure the polarization of the alkali metal vapor and broadband optical absorption spectroscopy to measure the vapor density-

### B. Transmission monitoring

Monitoring the pump beam transmission through the cell can be used as an alternative method to measure the alkali atom polarization.^12^ This method is also referred to as the field cycling method.^2,13,14^ As pointed out by Kelley and Branca,^2^ this method is limitedin that it can only be used when the attenuation of laser light in the optical pumping cell obeys Beer’s law. Here, again we state the reasoning. The attenuation of the photon flux of the pump laser down the length of the optical cell is given by

$$\frac{d\phi }{dz}=-\left[Rb\right]\left(1-P_{Rb}\left(z\right)\right)\gamma_{p}\left(z\right),$$ (3)

where *γ*_*p*_ is the optical pumping rate. The optical pumping rate is proportional to the photon flux, *ϕ*, and the photon absorption coefficient beta, *β*,

$$\gamma_{p}=\beta \phi .$$ (4)

Therefore,

$$\frac{d\gamma_{p}}{dz}=-\left[Rb\right]\beta \left(1-P_{Rb}\left(z\right)\right)\gamma_{p}\left(z\right),$$ (5)

where [*Rb*]*β*(1 − *P*_*Rb*_(*z*)) is the position dependent absorption length.^15–17^ If we can treat the absorption length as relatively constant, the solutions to Eqs. (3) and (5) are equivalent to Beer’s law.

Assuming the pump laser light is 100% circularly polarized, the alkali polarization will be given by

$$P_{Rb}\left(z\right)=\frac{\gamma_{p}\left(z\right)}{\gamma_{p}\left(z\right)+\Gamma_{SD}},$$ (6)

where Γ_*SD*_ is the spin-destruction rate of Rb. The optical pumping rate will therefore obey

$$\frac{d\gamma_{p}\left(z\right)}{dz}=-\beta \left[Rb\right]\gamma_{p}\left(z\right)\left(1-\frac{\gamma_{p}\left(z\right)}{\gamma_{p}\left(z\right)+\Gamma_{SD}}\right).$$ (7)

If the Rb density is relatively homogeneous throughout the optical cell, the solution to Eq. (7) becomes

$$\gamma_{p}\left(z\right)=\Gamma_{SD}W\left(\frac{\gamma_{p}\left(0\right)}{\Gamma_{SD}}\exp\left(\frac{\gamma_{p}\left(0\right)}{\Gamma_{SD}}-\beta \left[Rb\right]z\right)\right),$$ (8)

where *W* is the LambertW function. In addition, if

$$\frac{\gamma_{p}\left(0\right)}{\Gamma_{SD}}\gg \beta \left[Rb\right]z,$$ (9)

the Rb polarization will not attenuate significantly down the cell and to the first order *P*_*Rb*_(*z*) ≈ *P*_*Rb*_(0). In this case, Eq. (3) reduces to

$$\frac{d\phi }{dz}=-\left[Rb\right]\left(1-P_{Rb}\left(0\right)\right)\beta \phi \left(z\right),$$ (10)

whose solution

$$\phi \left(z\right)=\phi_{o}\exp\left(-\left[Rb\right]\left(1-P_{Rb}\left(0\right)\right)\beta z\right)$$ (11)

is nothing else than Beer’s law. A more in depth discussion of the validity of the approximations made here is provided in the Appendix.

Due to the birefringent nature of the vapor, only those atoms in the *m*_*j*_ = 1/2 state can absorb the pump laser light for *σ*^−^. Similarly, only atoms in the *m*_*j*_ = −1/2 state can absorb *σ*^+^ light. Assuming a *σ*^+^ pump beam, one can express the alkali polarization as

$$\left\langle P_{Rb}\right\rangle =\frac{\left\langle N^{+}\right\rangle -\left\langle N^{-}\right\rangle }{\left\langle N^{+}\right\rangle +\left\langle N^{-}\right\rangle }.$$ (12)

We can then define the absorbance, *A*, as

$$A=\left[Rb\right]\left(1-P_{Rb}\right)\beta l=2\left\langle N^{-}\right\rangle \beta l,$$ (13)

where *l* is the path length and ⟨*N*^−^⟩ is the number density of alkali atoms in the *m*_*j*_ = −1/2 state. When the magnetic field is turned off and any residual field is orthogonal to the pump beam, the alkali polarization is approximately zero, and

$$A_{0}=\left[Rb\right]\beta l=\left(\left\langle N^{+}\right\rangle +\left\langle N^{-}\right\rangle \right)\beta l.$$ (14)

This means we can express the ratio of the absorbance with (*A*) and without (*A*_0_) the magnetic field as

$$\frac{A}{A_{0}}=\frac{2\left\langle N^{-}\right\rangle }{\left\langle N^{+}\right\rangle +\left\langle N^{-}\right\rangle }.$$ (15)

Manipulating this in a clever way allows us to extract the alkali polarization,

$$\begin{array}{ll} 1-\frac{A}{A_{0}} & =\frac{\left\langle N^{+}\right\rangle +\left\langle N^{-}\right\rangle }{\left\langle N^{+}\right\rangle +\left\langle N^{-}\right\rangle }-\frac{2\left\langle N^{-}\right\rangle }{\left\langle N^{+}\right\rangle +\left\langle N^{-}\right\rangle }=\frac{\left\langle N^{+}\right\rangle -\left\langle N^{-}\right\rangle }{\left\langle N^{+}\right\rangle +\left\langle N^{-}\right\rangle }\\ & =\left\langle P_{Rb}\right\rangle , \end{array}$$ (16)

and finally,

$$\left\langle P_{Rb}\right\rangle =1-\frac{A}{A_{0}}.$$ (17)

Note that if *σ*^−^ is instead assumed, Eq. (17) becomes $\left\langle P_{Rb}\right\rangle =\frac{A}{A_{0}}-1$. In the case of a pressure broadened vapor and narrow pump beam (i.e., when the linewidth of the laser is narrower than the absorption linewidth of the vapor), one may simply use a photodiode and relate the voltage to the transmittance *T*,

$$A=-\ln\left(T\right)=-\ln\left(\frac{V}{V_{c}}\right),$$ (18)

where V is the voltage with vapor and *V*_*c*_ is that without vapor (i.e., for a cool cell). Finally, we have that

$$\left\langle P_{Rb}\right\rangle =1-\frac{\ln\left(\frac{V}{V_{c}}\right)}{\ln\left(\frac{V_{0}}{V_{c}}\right)},$$ (19)

where *V*_0_ is the photodiode voltage in the absence of a magnetic field.

In the case where the alkali absorption peak is narrower than the pump beam, one must use an optical spectrometer to carefully integrate the overlapping peaks.^13,18^ In addition, in the presence of stray magnetic fields along the direction of the pump beam, this measurement will be an underestimation of the true polarization. Therefore, it is important to either align the system perpendicular to Earth’s magnetic field or use a secondary pair of coils (active shimming) or removable permanent magnets (passive shimming) to null the stray field. If choosing to align perpendicular to Earth’s magnetic field and forgo shimming, as we did, one must ensure that the resultant skew angle is minimized.^19^ For our 15 G pump field, Earth’s magnetic field led to a negligible skew angle of <2°. Furthermore, this method does not account for the Rb polarization outside of the pump beam, that is, near the walls where the Rb polarization goes to zero. Although it has its own limitations, the transmission monitoring method is substantially easier to implement on standard SEOP setups capable of producing liters of polarized gas at a time. In what follows, we are set to compare the accuracy of this method with respect to the Faraday rotation method.

## II. METHODS

### A. SEOP setup

**FIG. 1.**
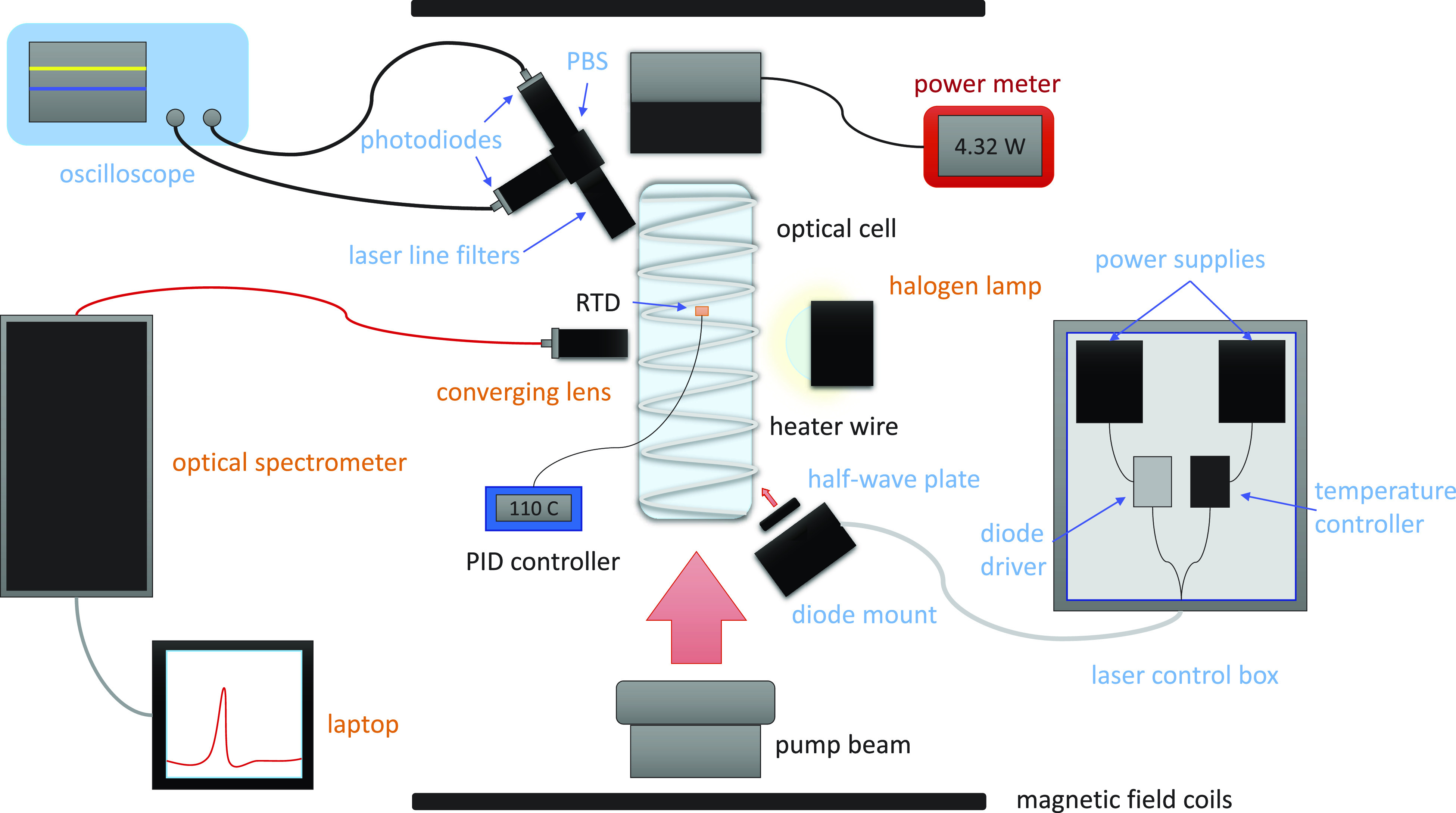
Experimental setup. Components necessary for Faraday rotation measurements are indicated in blue font. Red font indicates the equipment necessary for transmission monitoring. Orange font indicates equipment needed for optical absorption measurements. The equipment labeled in black font is necessary for optical pumping.

Measurements were made on a lab built polarizer equipped with a BrightLock pump laser with a 795 nm center wavelength and a 0.18 nm linewidth capable of producing up to 170 W (QCP Lasers, Sylmar, CA). The laser had a temperature tuning coefficient of ∼0.07 nm/°C. Laser wavelength tuning was repeated for each injection current using a water chiller to ensure a resonance condition. The laser power was measured using a Thorlabs (Newton, NJ) thermal power sensor (S322C/PM100D). The magnetic field (15 G) was produced using electromagnetic coils in a Lee-Whiting arrangement (Acutran Fombell, PA) powered by a constant current power supply (TDK-Lambda Neptune, NJ). All measurements were performed on a small (with a 5 cm diameter and a length of 15 cm) optical cell filled with 4.7 atm (3.4 amg) of 2/10/88 of Xe/N_2_/He. To gain access to all sides of the optical cell, the cell was heated using a heater wire wrapped around the main body of the cell. The cell surface temperature was controlled and maintained using a proportional–integral–differential (PID) controller and a resistance temperature detector (RTD) probe. As the wire produced a small magnetic field along the long axis of the cell, measurements were taken only when the heating coil current was turned off. The experimental setup can be seen in Fig. 1. 

**FIG. 2.**
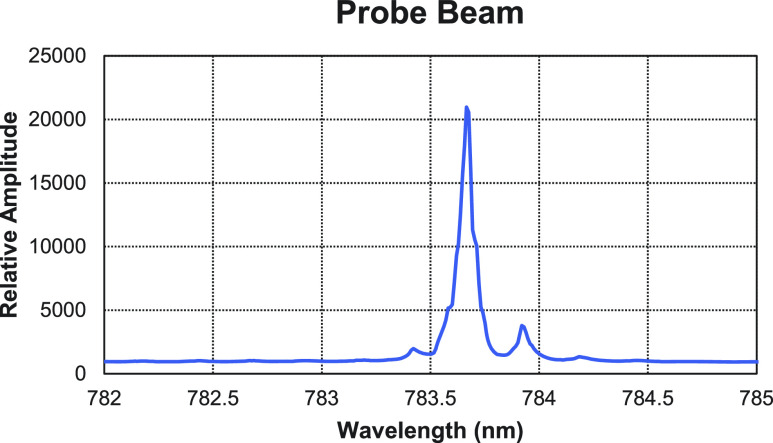
Probe beam as measured by our lab built optical spectrometer. Almost single mode lasing was achieved. As the probe beam is far detuned from resonance, the minor mode does not pose any significant contribution to the rotation angle. Full details of the optical spectrometer can be found in the supplementary material of the study by Antonacci *et al.*^20^

### B. Broadband optical absorption spectroscopy

The Rb vapor density inside the optical cell was measured by broadband optical absorption spectroscopy using a 20 W halogen bulb with a condenser lens, a 100 mm focal length lens, and our lab built optical spectrometer with a resolution of 0.009 nm per pixel. According to Beer’s law, the intensity of light passing through an absorbing medium can be described as

$$I\left(\nu \right)=I_{o}\left(\nu \right)\exp\left(-\left[Rb\right]l\sigma \right),$$ (20)

where *I*_*o*_ is the characteristic line shape of the light source (i.e., the spectra without the absorbing medium), *I* is the intensity of light after passing through the sample of length *l*, and *σ* is the absorption cross section. By rearranging Eq. (20) and integrating over the frequency, one can write the Rb vapor density as

$$\left[Rb\right]=\frac{1}{\pi r_{o}cfl}\int \ln\left(\frac{I_{o}\left(\nu \right)}{I\left(\nu \right)}\right)d\nu ,$$ (21)

and by definition,

$$\int \sigma \left(\nu \right)d\nu =\pi \;r_{o}\;c\;f,$$ (22)

where *r*_*o*_ is the classical electron radius, *c* is the speed of light, and *f* is the oscillator strength. The absorbance, ln(*I*/*I*_*o*_), can be fit to a modified Lorentzian,

$$L\left(\nu \right)=\frac{A+2\pi T\left(\nu -\nu_{o}\right)}{{\left(\nu -\nu_{o}\right)}^{2}+{\left(\frac{\gamma }{2}\right)}^{2}}+B+C\left(\nu -\nu_{o}\right),$$ (23)

where *A* is the amplitude, *T* is the asymmetry parameter, *γ* is the linewidth, and *B* and *C* represent unphysical gains. Then finally, the density can be calculated as

$$\left[Rb\right]=\frac{2A}{\gamma \;\times r_{o}\times c\times \;f\times \;l}.$$ (24)

We used the pressure dependence reported by Romalis *et al.*^21^ to calculate the *D*_2_ oscillator strength, $f_{D_{2}}=0.62$. For the oscillator strength of the *D*_1_ transition, for which they did not report a pressure dependence, we used their value of 0.33. To correct for the cylindrical shape of the optical cell and therefore the non-uniform path length, the following expression was used:

$$l=\frac{1}{r}\int_{-r}^{r}\sqrt{R^{2}-u^{2}}du,$$ (25)

where *R* is the radius of the optical cell and *r* is the radius of the 100 mm focal length lens.

### C. Faraday rotation setup

In order to perform Faraday rotation measurements, there are two main components required—a low power probe laser that can be detuned from the resonances of the alkali metal and a polarization detector. Many previous experiments utilize expensive laser systems, such as pumped Ti:Sapphire lasers.^8,22–25^ However, we wished to put together a low-cost system with a small footprint that could be used aboard most of the SEOP polarizers used for the production of large quantities of hyperpolarized gas without significant modifications. Small, low-power laser diodes with a center wavelength around 780 nm suitable to probe the *D*_2_ of Rb are quite affordable. By using a temperature-controlled mount, these diodes can be stabilized and tuned across several nm. For our measurements, we used a 10 mW 780 nm laser diode (L780P010) in a commercial laser diode mount from Thorlabs (TCLDM9/LDM21), along with a low noise diode driver (MPL250) and temperature controller (PTC2.5K-CH 2.5A) from Wavelength Electronics (Bozeman, MT), which were powered by linear power supplies from Bel Power Solutions (HAA15-0.8/HB5-3 Santa Clara, CA).

Before aligning the probe laser with the detector, a lab-built optical spectrometer was used to monitor the probe beam as a function of injection current and temperature. The current was first adjusted to get nearly single mode operation. Then the temperature was adjusted to tune the probe beam off resonance by ≈4 nm. This calibration step was important as any absorption of the probe would result in an apparent reduction of the rotation angle. The linewidth of the probe beam was 0.08 nm and was centered at 783.67 nm (Fig. 2). As the probe beam wavelength was detuned by several nm from the pressure broadened *D*_2_ resonance (linewidth ≈ 0.25 nm), it did not require further linewidth narrowing using a cavity and was treated as monochromatic. A half-wave plate in a rotation mount (Thorlabs WPMH05M-780, RSP1) was used to rotate the initial polarization of the probe beam to optimize the measurement.

For the detector, we choose the design of a balance polarimeter. We used a cage mounted polarizing beam splitter (Thorlabs CCM5-PBS202) with several 780 nm laser line filters (FL05780-10) to block the pump beam along with two mounted photodiodes (SM05PD3A). The output of these photodiodes was measured using a dual channel oscilloscope. Full details and parts list can be found in the supplementary material.

The probe laser was aligned with the polarization detector to cover most of the optical cell. Because a Rb layer coats most of the back of the cell, we found that the optimum placement was at a *ϕ* ≈ 30° angle from the magnetic field and pump beam such that

$$P_{Rb}=\frac{6mc}{\left[Rb\right]le^{2}}\theta_{P}\sec\phi {\left(\frac{\Delta_{3/2}}{{\left(\Delta_{3/2}-\delta_{3/2}\right)}^{2}}-\frac{\Delta_{1/2}}{{\left(\Delta_{1/2}-\delta_{1/2}\right)}^{2}}\right)}^{-1}.$$ (26)

### D. Experimental protocol

Before the optical cell was heated, the pump beam was blocked, and the laser was allowed to come to the set base temperature, which was predetermined using our optical spectrometer to ensure resonance. This was to prevent the pump beam from heating the optical cell and altering the baseline measurement of the transmitted power. Once the pump laser was at temperature, the block was removed, and voltage was measured by the power meter at the back of the cell. The beam was blocked again, and the heater coil was then turned on and set to the operating temperature for the measurement.

To account for imperfections in the half-wave plate and different responsivities of the photodiodes, the maximum and the minimum voltages of photodiodes A and B (*U*_*A*_ and *U*_*B*_) were measured by rotating the waveplate. This was performed before and after each set of measurements when the optical cell was warm and the pump laser was blocked. To maximize the sensitivity of the measurement, the outputs of the photodiodes were approximately balanced using the half-wave plate such that the initial angle of the probe beam was ≈ 45°. The initial voltages were then recorded. For each measurement, we calculate the initial angle *θ*_*initial*_ (unpolarized Rb vapor) and the final angle *θ*_*final*_ (polarized Rb vapor) as

$$\theta =\arctan\sqrt{\frac{U_{A}-U_{A_{\min}}}{U_{A_{\max}}-U_{A_{\min}}}\times \frac{U_{B_{\max}}-U_{B_{\min}}}{U_{B}-U_{B_{\min}}}}.$$ (27)

The difference between the initial and final angle gave the Faraday rotation angle, *θ*_*F*_,

$$\theta_{F}=\theta_{\mathrm{final}}-\theta_{\mathrm{initial}}.$$ (28)

After these measurements, the pump beam was unblocked, and the cell temperature was allowed to stabilize for 10 min. Since we were working with relatively low pump laser powers (max of 40 W), there were no significant deviations in cell temperature during the measurement. Once the temperature was stabilized, we blocked the probe beam to measure the residual leakage from the pump beam that was not removed by the laser line filters. This pump leakage was only ≈1% of the total signal, but if not accounted for, it would have caused a drift in the observed rotation angle as the pump laser light was absorbed at higher densities. These values were subtracted from the signals measured during optical pumping. Note that by swapping the position of the probe beam and polarization detector, one could have slightly reduced the leakage signal from the pump beam. Unfortunately, this was not possible in our case due to constraints on the laser control box mounting of our setup. The voltages measured by the two photodiodes were recorded during optical pumping as well as the transmitted laser power measured at the back of the cell. The magnetic field was then turned off (the polarizer was oriented such that the residual Earth field was perpendicular to the pump beam), and the transmitted laser power was then recorded.

The magnetic field was then turned back on, and the procedure was repeated four more times. To reduce variability in the measurements, after the magnetic field was turned on, we waited until the transmitted laser power returned to its original value before repeating the measurement. After the fifth measurement, we turned off the field and immediately took the absorption spectra to measure the Rb density. 500 averages were taken in less than 5 s after the laser was turned off. These measurements were repeated for different cell temperatures. After they were completed, the pump beam was again blocked, the cell was allowed to cool down, and the cool cell absorption spectra were taken. Then pump beam was unblocked, and the transmitted power was recorded again. Two separate sets of measurements were taken—one with the probe beam going through the first half of the optical cell and a second set where the probe beam went through the entire optical cell at 24 and 40 W of pump power. To ensure that the removal of laser heating did not alter the density measurement, we compared the first absorption spectrum to the last and found no decrease in the density measured between the two, indicating that the measurements were stable and representative of the density during optical pumping. Further testing showed a fairly stable Rb density (<3% change) even 50 s after the removal of the pump laser. Finally, to ensure that a homogeneous vapor density could be assumed, we also measured the Rb density across several points in the cell and found a <10% deviation in the values. This check was performed to ensure that a homogeneous vapor density could be assumed.

## III. RESULTS

**FIG. 3.**
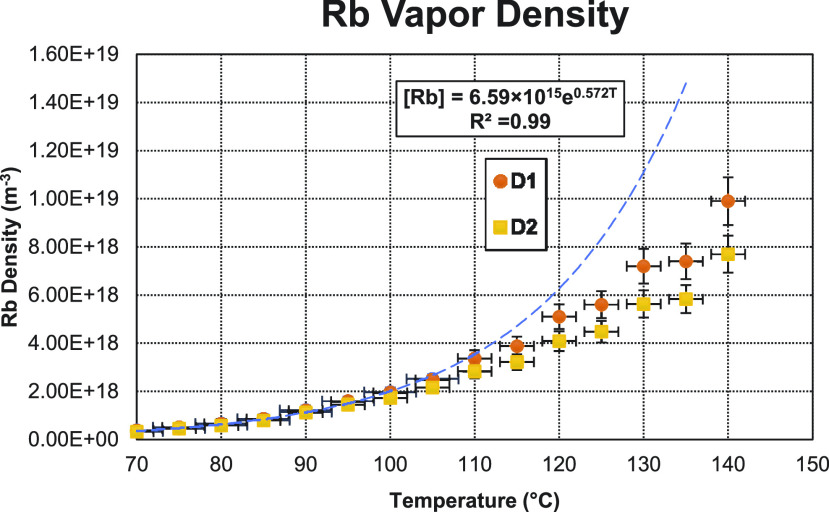
Example of the broadband optical spectroscopy measurements of the Rb density. The orange circular data points represent the density as measured by the *D*_1_ peak. The yellow square data points represent the density as measured by the *D*_2_ absorption peak. Note how after ≈105 °C the *D*_2_ measurements (yellow squares) begin to predict a lower density that are then followed by the *D*_1_ measurements (orange circles). The blue dashed line represents the fitting of the data where Beer’s law holds. Note how the data points that violate Beer’s law (i.e., above ≈115 °C in this case) show a linear increase with temperature instead of the expected exponential increase.

The caveat with broadband optical absorption spectroscopy is that the medium cannot be “optically thick,” here defined as when the transmittance is less than 1/*e* or ≈37%. This condition is easy to satisfy when working at high gas pressures (4.7 atm), which broadens the resonances, and relatively low temperatures. However, at the highest temperatures we used, deviation from Beer’s law could be seen, first in the case of the *D*_2_ transition, which has an oscillator strength approximately twice that of the *D*_1_ transition, and then finally in the *D*_1_ transition. To compensate for this effect, we fit the density vs temperature data where Beer’s law is applicable and extrapolate the points where the medium becomes optically thick. This process was repeated for every group of measurements. An example is shown in Fig. 3. Note that those higher temperature data points increase linearly with temperature instead of exponentially, a clear indication that Beer’s law is violated.

**FIG. 4.**
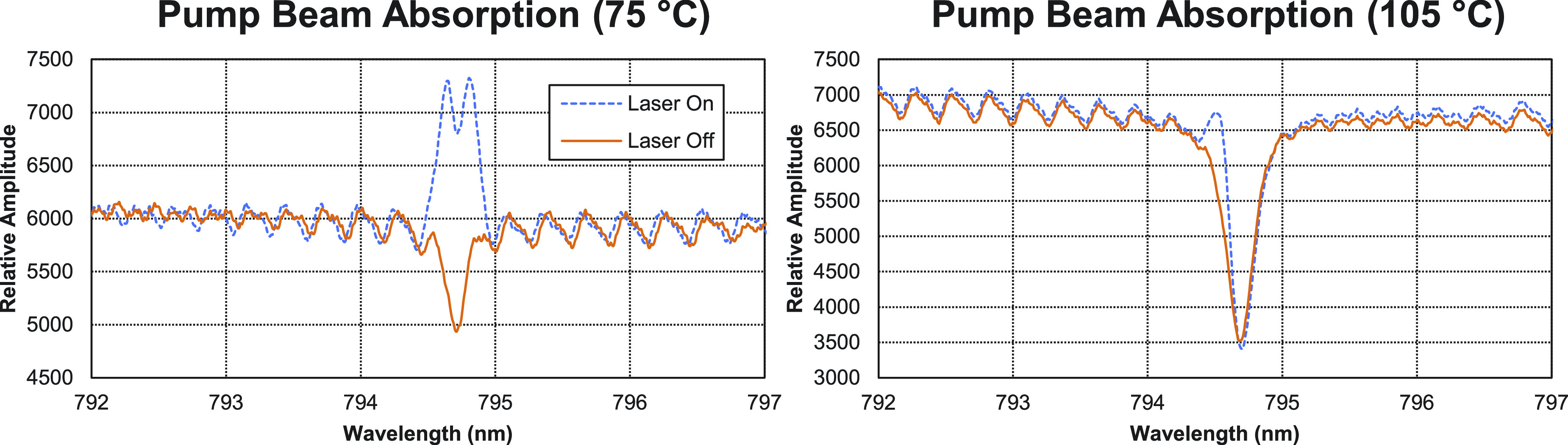
The dashed blue line represents the reflected, orthogonal pump laser light, which is being absorbed by the Rb vapor. The solid orange line represents the *D*_1_ absorption peak (i.e., halogen lamp and no pump laser). Comparing the two temperatures, it is easy to see that the laser is on resonance and all pump light is able to be absorbed, which is an important check for transmission monitoring.

Figure 4 shows the relative amount of pump light absorbed by the polarized vapor. The solid orange “laser off” curve represents the absorption spectra of *D*_1_ taken using the halogen lamp. The dashed blue curve, “laser on,” represents the amount of laser light absorbed. Note that this is the reflected light, measured perpendicular to the pump beam. From this, we can see that the pump beam is centered on *D*_1_. Furthermore, at the higher temperature, almost all the laser light has been absorbed. This is an important check to ensure that all laser light can be absorbed, and thus, there is no distortion in the transmission measurements of the polarization.

**FIG. 5.**
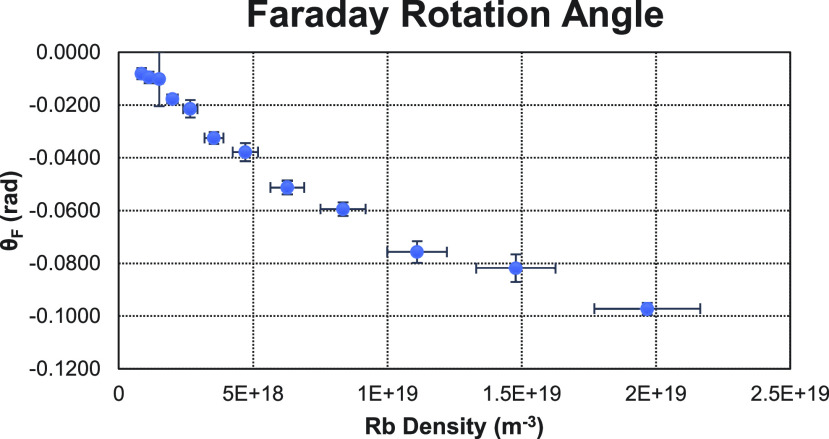
Example of the Faraday rotation angles measured with a 40 W pump beam.

**FIG. 6.**
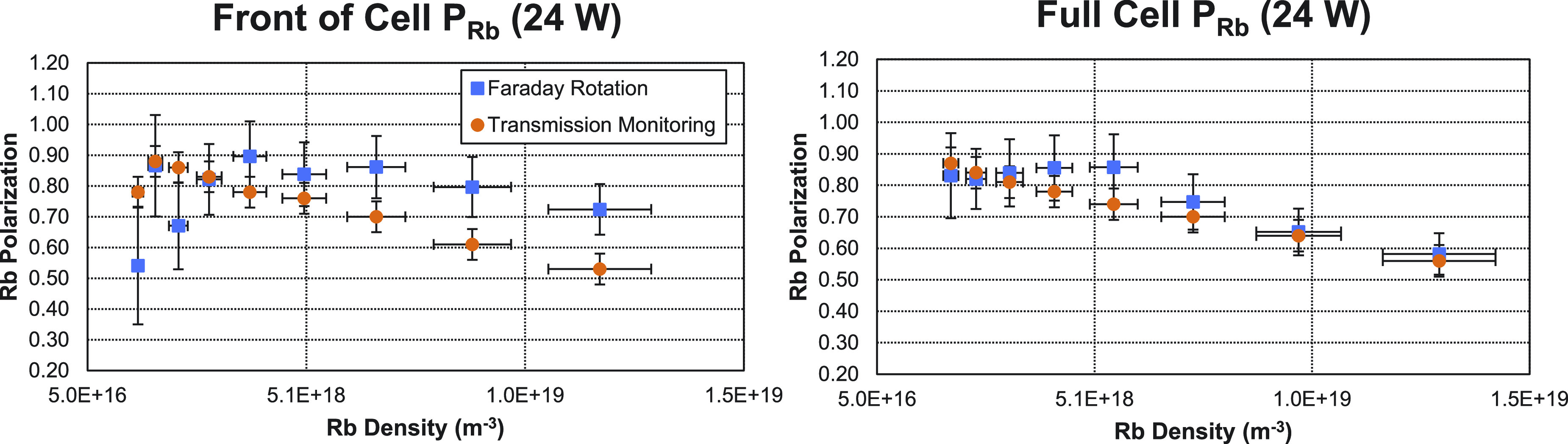
Comparison of the two methods of measuring the Rb polarization with 24 W of pump power. The blue square data points represent the Faraday rotation measurements, and the orange circles represent the transmission monitoring data points. On the left, the Faraday rotation measurements were only taken through the front half of the optical cell. On the right, the Faraday rotation measurements were taken through the full cell. As the Rb density increases, note how the Faraday rotation measurements made at the front of the cell overestimate the Rb polarization compared to measurements made by transmission monitoring over the entire cell.

**FIG. 7.**
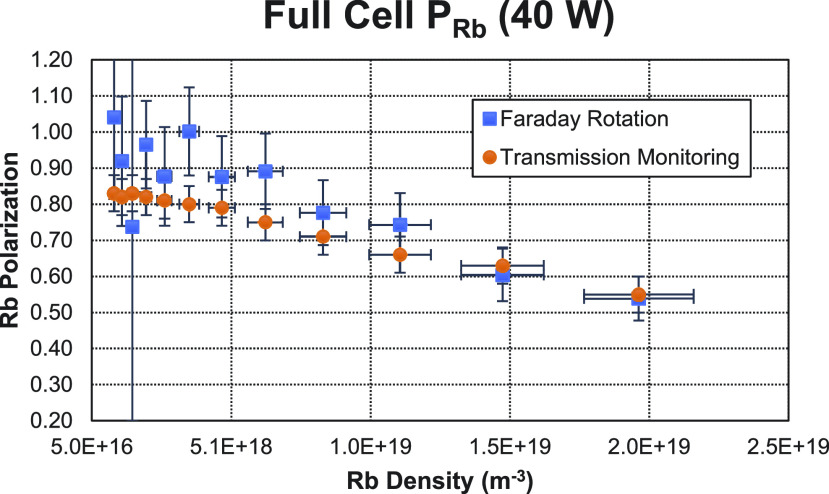
Comparison of measurements of the Rb polarization using Faraday rotation (blue squares) and transmission monitoring (orange circles) with 40 W of pump power.

Figure 5 shows an example of the measured Faraday rotation angles with 40 W of pump power as a function of Rb density. In Fig. 6, a comparison of the Rb polarization measured with Faraday rotation and with transmission monitoring is shown. On the left, the Faraday rotation measurements were taken through the first half of the optical cell. On the right, the Faraday rotation measurements were taken over the entire optical cell. In both cases, the transmission monitoring measurements were taken through the entire cell. Figure 7 shows additional measurements taken over the entire cell using 40 W of pump power.

## IV. DISCUSSION

### A. Density measurements

Due to the experimental limitations of working at relatively low magnetic field strengths and Rb densities, we were unable to detect the field dependent Faraday rotation to measure the Rb density. Instead, we used broadband optical absorption spectroscopy at the *D*_1_ and *D*_2_ lines. This method has limitations imposed by Beer’s law that the medium cannot be optically thick (i.e., transmission <1/*e*). Due to our pressure broadened conditions, our lower temperature measurements were not optically thick, and we were able to extrapolate the Rb densities for higher temperatures (Fig. 3). We acknowledge that extrapolating the density is not ideal; however, we wished to test methods that were accessible to those working with on polarizers with relatively large optical cells. At the highest temperature tested (140 °C), ignoring the deviations from Beer’s law would have resulted in a maximum of 55% underestimation of the Rb density. In comparison, if we were to assume thermodynamic equilibrium in the cell and use an empirical formula, as often done, we would have overestimated the Rb density by 200%.

For systems operating at lower pressures, broadband absorption spectroscopy of *D*_1_ and *D*_2_ will be challenged by the much narrower linewidths and increased maximum absorbance. However, these measurements may be feasible at the other persistent line of Rb, around 421 nm (6*p* → 5*s*), which has an oscillator strength two orders of magnitude smaller than *D*_1_ and *D*_2_. Under our pressure broadened conditions, the absorption peak at 421 nm was too small to be detected.

Finally, note that we did not see a deviation in the density measured during the first spectrum compared to the 500^th^; therefore, we do not believe that laser heating caused a significant change in the density during the time span between turning off the laser and acquiring the spectra.

### B. Rb polarization measurements

The Rb densities measured here were comparable to those previously found during SEOP.^2^ This, combined with the detuning necessary for our pressure broadened conditions, meant that our Faraday rotation angles were overall quite small, ranging from 5 to 100 mrads. At our lowest densities, this meant large errors in measured rotation angles, particularly for the measurements through the front half of the cell. In all cases (Figs. 6 and 7), it appears that the Rb polarization is constant over a short range of densities until the density is increased such that the polarization can no longer be maintained and begins to drop. It is at this same point where the Faraday rotation measurements begin to detect a higher polarization at the front of the optical cell (Fig. 6). This tells us that the Rb polarization is dropping along the length of the optical cell, which is consistent with previous models and experimental measurements that have been made on similar setups.^1,26,27^

The measurements at 40 W (Fig. 7) show inconsistencies between the two methods at the lowest densities. Specifically, the Faraday rotation method gives a Rb polarization much higher than the one measured by the transmission monitoring method. We do not have reason to believe that the transmission monitoring method was failing at these densities as the experimental error on these data points was quite small. On the other hand, we believe that the high optical pumping rate, under the experimental conditions used here (low Rb density and high laser power), is leading to non-linear effects that arise from the strong light field of the pump beam.^9,28–31^ These sorts of effects have been documented for light fields of a few mW per mm^2^.^32^ For the 40 W beam, which at these low densities led to a transmission of 0.88 during optical pumping, the average light field throughout the cell was 18 mW per mm^2^. In particular, Budker *et al.*^31^ noted that upper state saturation could result in nonlinear effects that would alter the paramagnetic Faraday rotation. We believe that this is what is being observed at these high optical pumping rates, but the resolution of our setup did not allow us to further explore this beyond conjecture. Nonetheless, because the transmission monitoring method is only sensitive to the ground state polarization, there is no reason to believe that these effects would affect these measurements.

Overall, we find good agreement in the Rb polarization values obtained with the Faraday rotation method and those obtained with the transmission monitoring method, with the latter being by far the preferable method for measuring the alkali metal polarization on our SEOP setup. Indeed, transmission monitoring requires fewer optical components and does not require the meticulous calibration needed for the Faraday rotation measurements. On pressure broadened systems with the latest generation of spectrally narrowed lasers, a power meter suffices to measure the polarization. For lower pressure systems, we suggest constructing an optical spectrometer such as the one we employed^20^ and integrating the spectral overlap as carried out by Nikolaou.^13^ We initially had concerns that as transmission monitoring assumes that Beer’s law can be used to describe the attenuation of light down the optical cell, it would not be valid for low polarization values. However, we found good agreement between the two methods down to *P*_*Rb*_ = 0.5. Perhaps, at the very lowest Rb polarization values, transmission monitoring would not be applicable, but for polarization values typically obtained during the production of hyperpolarized^129^Xe, this method is valid. Furthermore, we would like to note that the combination of transmission monitoring and Faraday rotation could also be used to measure the Rb density. This has potential for those systems where broadband optical absorption spectroscopy cannot be carried out (i.e., those in which the medium is optically thick). Finally, our data and the literature seem to indicate that there are non-linear effects in strong light fields that may prevent the use of the Faraday rotation method under the conditions commonly used for SEOP. This deserves further inspection with higher resolution methods than those used here.

## SUPPLEMENTARY MATERIAL

Detailed instructions for the construction of the diode laser and polarimeter are provided in the supplementary material, along with a bill of materials.

## Figures and Tables

**FIG. 8.**
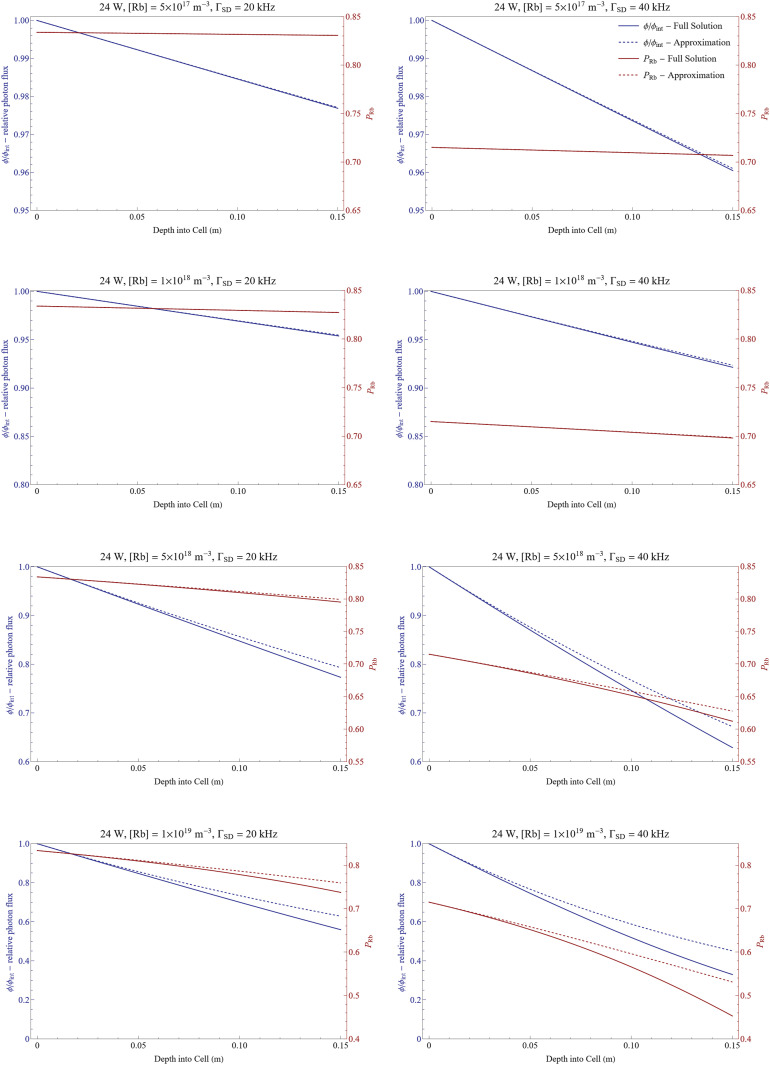
Relative photon flux (left blue axis) and Rb polarization (right red axis) as a function of depth into the optical cell for 24 W of pumping power. Our approximation (dashed curves) closely matches the full solution (solid curves) across all conditions tested except for the combination of the highest Rb density (1 × 10^19^ m^−3^) and the highest spin-destruction rate, 40 kHz (bottom right figure). In this case, $\gamma_{\bar{\left(z\right)}}/\Gamma_{SD}=1.6$.

**FIG. 9.**
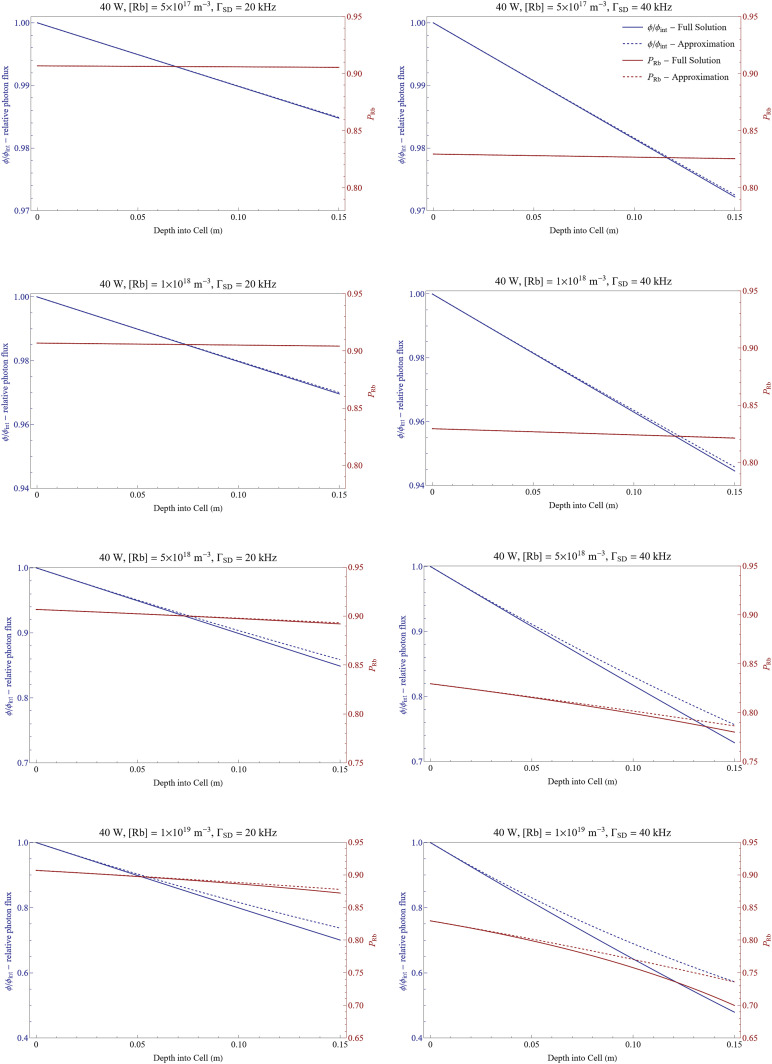
Relative photon flux (left blue axis) and Rb polarization (right red axis) as a function of depth into the optical cell for 40 W of pumping power. Our approximation (dashed curves) closely matches the full solution (solid curves).

## Data Availability

The data that support the findings of this study are available within the article and its supplementary material.

## APPENDIX: TRANSMISSION MONITORING

In Sec. I B, we showed that under the assumption $\frac{\gamma_{p}\left(0\right)}{\Gamma_{SD}}\gg \beta \left[Rb\right]z$, the absorption length *β*[*Rb*](1 − *P*_*Rb*_(*z*)) is approximately constant, which greatly simplifies the solution for the photon flux, from a LambertW function to an exponential decay, *ϕ*(*z*) = *ϕ*_*o*_ exp(−[*Rb*](1 − *P*_*Rb*_(0))*βz*), that is, Beer’s law.

Here, we analyze the validity of this assumption under the experimental conditions used in this work. Specifically, using the same formalism found in the study by Appelt *et al.*,^26^ we compute the photon flux and the resulting Rb polarization along the optical cell for the full solution and our approximation. Recall that the full solution for the optical pumping rate is

$$\gamma_{p}\left(z\right)=\Gamma_{SD}W\left(\frac{\gamma_{p}\left(0\right)}{\Gamma_{SD}}\exp\left(\frac{\gamma_{p}\left(0\right)}{\Gamma_{SD}}-\beta \left[Rb\right]z\right)\right),$$ (A1)

which gives a photon flux

$$\phi_{p}\left(z\right)=\frac{\Gamma_{SD}}{\beta }W\left(\frac{\gamma_{p}\left(0\right)}{\Gamma_{SD}}\exp\left(\frac{\gamma_{p}\left(0\right)}{\Gamma_{SD}}-\beta \left[Rb\right]z\right)\right).$$ (A2)

Equation (A2) was computed numerically in Mathematica (Wolfram Research Princeton, NJ). The computation was carried out for the two laser powers used in this work (24 and 40 W) for four different Rb densities (0.5, 1, 5, and 10 × 10^18^ m^−3^) to cover the density range measured in our experiments and for two different spin destruction rates. For the spin-destruction rate, we used the value of 20 kHz taken from the study by Appelt *et al.*^26^ and Norquay *et al.*^16^ and included a much higher spin-destruction rate of 40 kHz to fully probe the solution set. For our two laser powers, 24 and 40 W, we find *β* to be 1.86 × 10^−18^ and 2.18 × 10^−18^ m^2^, respectively, and *γ*(0) to be 100352 and 194634 Hz, respectively. The linewidths used in calculating these parameters were measured using our optical spectrometer.

Figures 8 and 9 show the computed relative photon flux and Rb polarization values for 24 and 40 W of laser power, respectively. These plots show that under all experimental conditions used here, the approximation $\phi \left(z\right)=\phi_{o}\exp\left(-\left[Rb\right]1-P_{Rb}\left(0\right)\beta z\right)$, which is at the core of the transmission monitoring method, produces reasonable photon fluxes and Rb polarization values. At the same time, these computations show that for very high Rb densities, lower laser powers, and longer optical cells, such approximations may no longer produce accurate photon fluxes and lead to an overestimation of the Rb polarization values, as one would expect.
